# P-510. Describing the Cohort of Women Veterans Prescribed HIV PrEP at the Veterans Health Administration

**DOI:** 10.1093/ofid/ofae631.709

**Published:** 2025-01-29

**Authors:** Kaitlyn Broderick, Shimrit Keddem, Lauren Beste, Anneliese Sorrentino, Tammara Dixon, Aneeza Agha, Omaris Caceres

**Affiliations:** University of Pennsylvania Perelman School of Medicine, Decatur, Georgia; Michael J. Crescenz VA Medical Center, Philadelphia, Pennsylvania; VA Puget Sound Health Care System, Seattle, Washington; Corporal Michael J. Crescenz Veterans Affairs Center for Health Equity, Research & Promotion (CHERP), Philadelphia, PA, Philadelphia, Pennsylvania; Corporal Michael J. Crescenz Veterans Affairs Center for Health Equity, Research & Promotion (CHERP), Philadelphia, PA, Philadelphia, Pennsylvania; Corporal Michael J. Crescenz Veterans Affairs Center for Health Equity, Research & Promotion (CHERP), Philadelphia, PA, Philadelphia, Pennsylvania; Corporal Michael J. Crescenz Veterans Affairs Center for Health Equity, Research & Promotion (CHERP), Philadelphia, PA, Philadelphia, Pennsylvania

## Abstract

**Background:**

Pre-Exposure Prophylaxis for HIV (PrEP) is a highly effective option available to women in the Veteran’s Health Administration (VHA). However, PrEP uptake among VHA patients has remained modest, particularly among women. To better understand PrEP uptake for women Veterans, we used VHA administrative data to describe the cohort of women Veterans prescribed PrEP and their prescribers at the VHA.

**Methods:**

We conducted a cross-sectional study of women enrolled in VHA care between January 1, 2012 and June 30, 2022 who were prescribed HIV PrEP for at least 30 days.

**Results:**

PrEP initiation increased gradually over the study years, with most patients receiving initial prescriptions after January 1, 2020. Among the cohort of 417 women, 48% lived in the South, and most were aged 18-39 years. In terms of racial demographics, 53% identified as white and 40% as Black, while ethnically Hispanic women constituted 12% of the group. Eleven percent were homeless. Nearly half of the cohort were noted to have military sexual trauma. There was a high prevalence of mental health disorders, with 85% experiencing depressive disorder. Regarding prescriber profiles, 22% were advanced practice providers, 20% were clinical pharmacists, and 58% were physicians, 87% of whom specialized in infectious diseases.

**Conclusion:**

These findings indicate an increase in prescriptions over time and significant geographic disparities among a vulnerable population of women Veterans. Although most patients lived in the South, this region has a lower rate of PrEP prescription to HIV incidence than in other regions. Most prescribers were infectious disease physicians, in contrast to estimates amongst general and commercially insured populations. Many patients in the study were prescribed PrEP via clinical pharmacists, and although data is lacking to compare to the general population, this suggests that Clinical Pharmacist Practitioners, who possess a scope of practice within the VHA which includes medication prescriptive authority and serving as a direct care provider, are an important tool to enhance PrEP uptake among women Veterans. More research is needed to identify barriers and strategies to overcome them for PrEP service implementation among women Veterans.

**Disclosures:**

**All Authors**: No reported disclosures

